# Chromatin interaction maps reveal genetic regulation for quantitative traits in maize

**DOI:** 10.1038/s41467-019-10602-5

**Published:** 2019-06-14

**Authors:** Yong Peng, Dan Xiong, Lun Zhao, Weizhi Ouyang, Shuangqi Wang, Jun Sun, Qing Zhang, Pengpeng Guan, Liang Xie, Wenqiang Li, Guoliang Li, Jianbing Yan, Xingwang Li

**Affiliations:** 1 National Key Laboratory of Crop Genetic Improvement, Huazhong Agricultural University, 1 Shizishan Street, Hongshan District, Wuhan, 430070 Hubei China; 2 Hubei Key Laboratory of Agricultural Bioinformatics and Hubei Engineering Technology Research Center of Agricultural Big Data, Huazhong Agricultural University, 1 Shizishan Street, Hongshan District, Wuhan, 430070 Hubei China

**Keywords:** Agricultural genetics, Epigenomics, Epigenomics, Transcriptional regulatory elements

## Abstract

Chromatin loops connect regulatory elements to their target genes. They serve as bridges between transcriptional regulation and phenotypic variation in mammals. However, spatial organization of regulatory elements and its impact on gene expression in plants remain unclear. Here, we characterize epigenetic features of active promoter proximal regions and candidate distal regulatory elements to construct high-resolution chromatin interaction maps for maize via long-read chromatin interaction analysis by paired-end tag sequencing (ChIA-PET). The maps indicate that chromatin loops are formed between regulatory elements, and that gene pairs between promoter proximal regions tend to be co-expressed. The maps also demonstrated the topological basis of quantitative trait loci which influence gene expression and phenotype. Many promoter proximal regions are involved in chromatin loops with distal regulatory elements, which regulate important agronomic traits. Collectively, these maps provide a high-resolution view of 3D maize genome architecture, and its role in gene expression and phenotypic variation.

## Abstract (web summary)

Spatial organization of regulatory elements and its impact on gene expression in plants remain unclear. Here, the authors construct maize chromatin interaction maps using chromatin interaction analysis by paired-end tag sequencing (ChIA-PET) and show their associations with gene expression and agronomic traits.

## Introduction

In eukaryotes, *cis* regulatory elements and associated *trans*-acting factors regulate spatiotemporal gene expression, which affects individual development. Remarkably, *cis* regulatory elements such as promoters, enhancers, and insulators have distinct epigenetic features^1^. For example, DNA methylation and histone modification play important roles in transcriptional regulation in both animals and plants^2–4^. In addition, extensive long-range chromatin interaction maps reveal that *cis* regulatory elements frequently form DNA loops mediated by specific proteins^5–8^. These structures, which include structures such as CTCF/cohesin-associated DNA loops, may coalesce into chromatin contact domains (also known as topologically associated domains), and further organize into active and inactive compartments as well as chromosome territories^9–11^. Accordingly, three-dimensional genome architecture models at various resolutions, based on chromatin interactomes, have provided a conceptual framework for transcriptional regulation in animals ranging from flies to humans^12^. Similarly, higher-order genome structures for *Arabidopsis*, rice, and other plants have been obtained via Hi-C technology^13–15^. However, due to the limited resolution of Hi-C maps, comprehensive high-resolution chromatin maps involving regulatory elements, which allow for elucidation of their impact on transcriptional regulation are still lacking in plants.

Maize is one of the most important crops. Many genetic variations outside protein-coding regions are associated with maize phenotypes. However, the mechanisms underlying these variations and their phenotypic expression are unclear. A few enhancers have been characterized in maize, including booster1 (*b1*)^16,17^, teosinte branched1 (*tb1*)^18,19^, vegetative to generative transition 1 (*vgt1*)^20,21^, and CCT transcription factor (*ZmCCT9*)^22^.

In this study, we explore the regulatory role of mutations occurring in distal regulatory elements related to gene expression and phenotypic variations with adapted long-read ChIA-PET, and construct high-resolution chromatin interaction maps of maize promoter proximal regions and distal regulatory elements associated with RNA polymerase II occupancy and histone mark H3K4me3. The resulting maps are analyzed to identify and assess the roles of promoter proximal–proximal interaction (PPI) and proximal–distal interaction (PDI). By integrating expression quantitative trait locus (eQTL) and genome-wide association study (GWAS) data, we demonstrate that long-range chromatin interactions between variant regulatory elements and their target genes contribute to variations in gene expression, metabolic phenotypes, and agronomic traits. Our results highlight the significance of 3D organization of regulatory elements and suggest that the topology of long-range genetic variations may affect gene expression as well as phenotype variation.

## Results

### Characterization of regulatory elements in maize seedlings

**Fig. 1:**
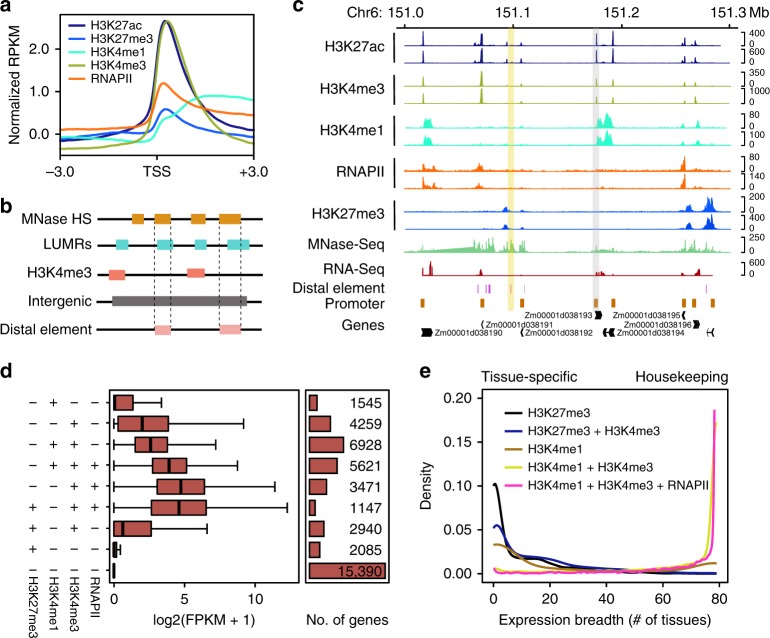
The epigenome map of maize seedlings. **a** Distribution of RNA polymerase II (RNAPII) occupancy and H3K4me3, H3K4me1, H3K27me3, and H3K27ac histone modifications at transcription start sites. **b** Illustration of candidate distal elements (MNase HS, MNase sequencing peak; LUMRs, low and unmethylated methylation regions). **c** RNAPII occupancy and H3K4me3, H3K4me1, H3K27me3, H3K27ac profiles on chromosome 6 (151–151.3 Mb). A promoter (gray shading) and a distal element (yellow shading) were highlighted in the region. **d** Gene expression levels corresponding to different combinations of RNAPII and histone modifications. Boxplots show the median, and third and first quartiles. The whiskers are defined as 0.25–1.5 IQR/0.75 + 1.5 IQR, IQR is interquartile range. **e** Expression breadth analysis of genes with different combinations of RNAPII and histone modifications. Source Data underlying (**d**, **e**) are provided in a Source Data file

We generated RNA Polymerase II (RNAPII) occupancy and histone modification profiles marked by H3K4me1, H3K4me3, and H3K27ac using ChIP sequencing in maize seedlings to map functional DNA elements and to investigate their regulatory role in gene expression. The data sets were reproducible between replicates, with Pearson correlation coefficients from 0.83 to 0.99 (Supplementary Fig. 1a and Supplementary Table 1). We identified 30,741 putative promoter proximal regions demarcated by H3K4me3 and H3K27ac marks (Fig. 1a and Supplementary Fig. 1c, d)^23^, along with 17,157 distal elements, which were identified as regions of accessible chromatin outside promoter proximal regions with lower levels of DNA methylation (Fig. 1b, c). Chromatin accessibility was obtained from published MNase sequencing data^24^. Strikingly, gene expression varied with different combinations H3K4me3, H3K27ac, and RNAPII occupancy, and especially genes marked with repressive H3K27me3 were expressed at significantly lower levels as expected (Fig. 1d and Supplementary Fig. 2a).

We further examined the expression patterns of genes with different epigenomic marks by using RNA-Seq data collected from 79 tissues^25^ (Supplementary Fig. 2b, c). Clustering analysis indicated that the expression of genes marked by H3K27me3 is mostly detected in a small number of tissues, suggesting that these genes may be tissue-specific, while genes marked by H3K4me1 and H3K4me3 were expressed in most tested tissues, indicating that they may be housekeeping genes (Fig. 1e and Supplementary Fig. 2d). These results highlight the synergistic effects of general histone modifications on gene expression patterns.

### Chromatin interaction associated with regulatory elements

**Fig. 2:**
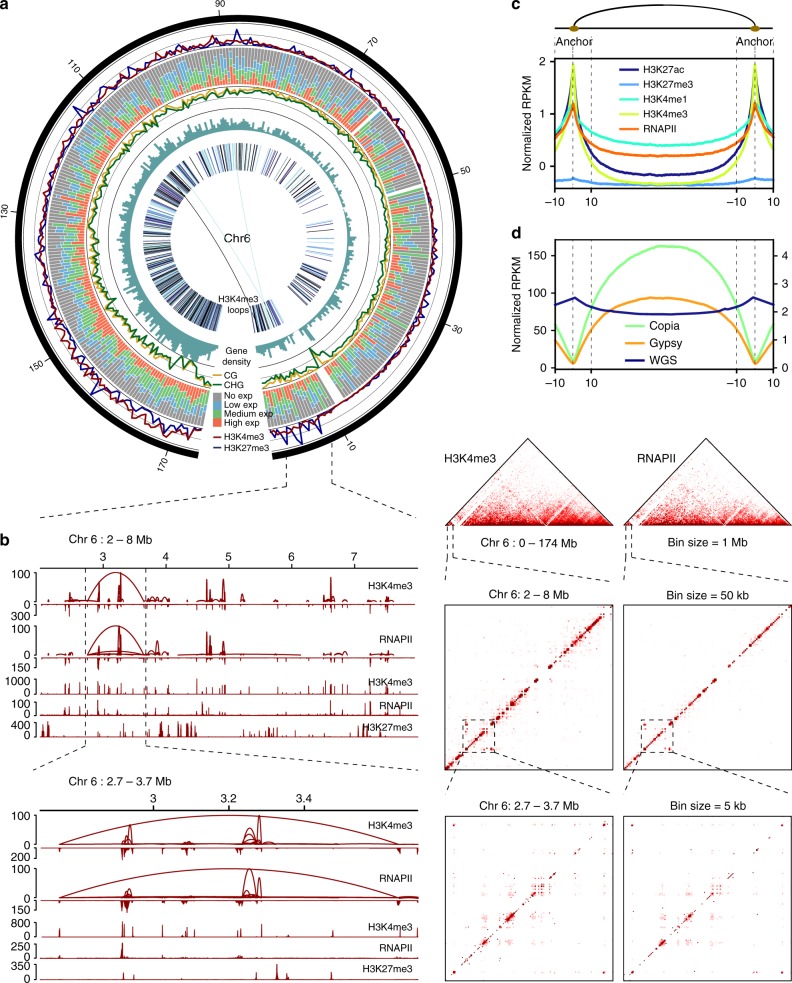
Characterization of RNAPII and H3K4me3 ChIA-PET data in maize seedlings. **a** Circos plot, with circles from outside to inside indicating histone modification (H3K4me3 and H3K27me3), gene expression (no expression, low expression, median expression, and high expression), DNA methylation (CG and CHG), gene density, and H3K4me3 interactions at 500 kb resolution on chromosome 6. **b** Detailed analysis of the 2–8 Mb region on Chr.6. Left panel: loop/peak view of H3K4me3 and RNAPII at indicated regions, with contact frequency in the loop or intensity of combined peaks indicated on the *y*-axis. Right panel: H3K4me3 and RNAPII contact heatmap. **c** Histone modifications, RNAPII occupancy, transposable elements, and MNase hypersensitivity around PPI anchors and loops. The *x* axis indicates a loop between anchors ± 10%, while the *y* axis indicates RPKM value. **d** Distribution of transposable elements Copia and Gypsy around PPI anchors and loops. *y*-Axis represents the normalized RPKM for the content of Copia and Gypsy (left) and whole genome sequencing (WGS) reads (right)

In order to map high-resolution long-range chromatin interactions associated with promoter proximal regions and regulatory elements, and examine their regulatory roles in gene expression, we applied the long-read ChIA-PET (chromatin interaction analysis by paired-end-tag sequencing) method to generate two independent datasets using RNAPII and H3K4me3 antibodies, respectively (Supplementary Fig. 3a, Supplementary Table 2, and Supplementary Data 1 and 2). Out of 49,766 H3K4me3 loops, we found 15,328 loops that both anchors are overlapped with H3K4me3 peaks and 34,438 loops with one anchor marked by H3K4me3 peaks; for 25,002 RNAPII mediated loops, 5692 loops have both anchors with RNAPII peaks and 19,310 loops have only one anchor associated with RNAPII peaks. ChIA-PET data provide not only high-resolution binding peaks and chromatin interaction loops of target proteins, but also high-resolution chromatin conformation^9^. Therefore, we first examined the contact frequency map at a 1 Mb resolution with our ChIA-PET data (Supplementary Fig. 3b). As illustrated in chromosome 6, chromatin interactions inferred from H3K4me3 were highly concurrent with histone modification enrichment, gene expression, and low DNA methylation (Fig. 2a). Similar patterns were also observed for the whole genome (Supplementary Fig. 4a and 4b). We next characterized high-resolution pairwise chromatin interactions inferred from H3K4me3-marked regions and RNAPII occupancy. We identified 28,875 PPI loops (Supplementary Data 3) based on 16,488,554 uniquely mapped paired-end tags from the combined RNAPII and H3K4me3 ChIA-PET data. These interaction loops connect 27,579 H3K4me3 peaks and 24,951 sites occupied by RNAPII, with examples illustrated at 2–8 Mb on chromosome 6 (Fig. 2b). Strikingly, we observed that intrachromosomal loops, both anchors of which are on the same chromosome, are much more frequent (~97%) than interchromosomal contacts (~3%), anchors of which are on different chromosomes (Supplementary Fig. 5). Furthermore, we found that interaction anchors were enriched with active epigenomic marks (including H3K4me3, H3K4me1, and H3K27ac) (Fig. 2c and Supplementary Fig. 6a) and were also strongly correlated with higher chromatin accessibility and lower DNA methylation level. In addition, these epigenomic features around the non-RNAPII and non-H3K4me3 anchor sites are similar with RNAPII and H3K4me3 anchors. Although the active histone marks are also enriched at non-RNAPII and non-H3K4me3 anchor regions, we observed the intensity summit of active histone marks shifted aside from the center of the non-RNAPII and non-H3K4me3 anchor regions (Supplementary Fig. 6b and 6c). In addition, anchor regions of chromatin interactions have lower transposable element contents especially for LTR-retrotransposon Copia (RLC) and LTR-retrotransposon Gypsy (RLG) than loop regions (Supplementary Fig. 6d and Fig. 2d). Together, our ChIA-PET data provide high-resolution chromatin interaction maps mediated by RNAPII and H3K4me3-marked regions to illuminate genome architecture.

### Genes with promoter proximal interaction are co-expressed

**Fig. 3:**
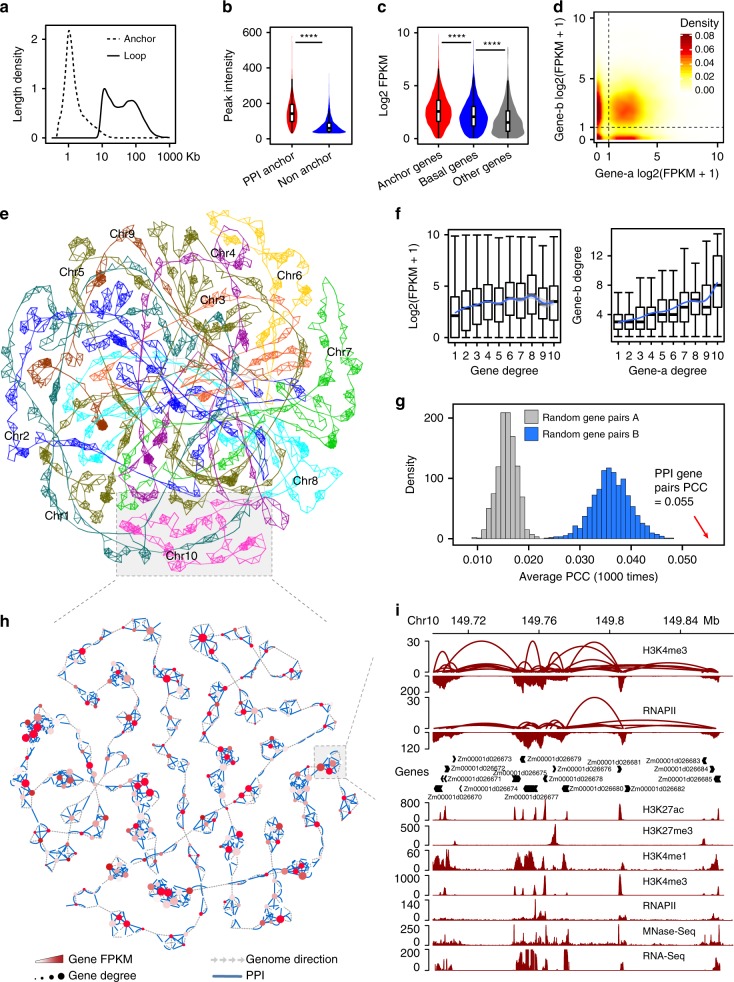
Characterization of promoter proximal–proximal interactions. **a** Span distribution of anchors and loops. **b** Intensity of anchor (*n* = 47,713) and non-anchor peaks (*n* = 8236). ****P < 0.0001 from Wilcoxon test. Boxplots show the median, and third and first quartiles. The whiskers are defined as 0.25–1.5 IQR/0.75 + 1.5 IQR, IQR is interquartile range. **c** Expression analysis of PPI anchor genes (*n* = 15,276), basal genes (*n* = 4959), and other genes (*n* = 877). Basal genes: genes containing H3K4me3 or RNAPII peaks but not involved in chromatin interaction; Other genes: genes neither marked with H3K4me3 or RNAPII peaks nor engaged in chromatin loops, ****P < 0.0001 from Wilcoxon test. Boxplots show the median, and third and first quartiles. **d** Contour map of log-transformed FPKM values obtained from RNA-Seq for genes involved in PPIs. **e** Self-organized maps of whole-genome PPI, with chromosomes colored individually. **f** Left: Gene expression as a function of the number of interactions. Right: Number of interactions of a gene in an interacting pair as a function of the number of interactions of the other gene in the same pair. Boxplots show the median, and third and first quartiles. **g** The average of Pearson correlation coefficients of expressions from PPI gene pairs is significantly higher than those from the random gene pairs. Random gene pairs A: random gene pairs from the same chromosome; random gene pairs B: random gene pairs with similar distance distribution and RNAPII or H3K4me3 peaks from PPI gene pairs. *P* < 0.0001 from *T*-test. **h** Representative subnetwork of intrachromosomal PPI in chromosome 10, as inferred from H3K4me3 (blue) and RNAPII occupancy (green). Directional dashed lines indicate linear connections. Node colors represent FPKM values obtained from RNA sequencing for genes involved in PPI, with lowest and highest values indicated in white and dark red, respectively. Node size indicates the number of connections with other nodes. **i** Examples of PPI. Top, intrachromosomal PPI inferred from H3K4me3 and RNAPII occupancy. Bottom, histone modifications, RNAPII occupancy, chromatin accessibility (MNase-Seq), and gene expression (RNA-Seq) at PPI sites. Source Data underlying (**b**–**d**, **f**, **g**) are provided in a Source Data file

Promoter proximal region interaction (PPI) loops generally span between 10 and 100 kb, and the width of anchor peak width is approximately at 1 kb (Fig. 3a). The intensity of anchor peaks, that associated with chromatin interactions, are generally higher than non-anchor peaks, which are not involved in chromatin interactions (*P* < 2.2e−16, Wilcoxon test, Fig. 3b). Out of 21,116 PPI anchor genes, 72.19% of them (15,276) are expressed (with FPKM > 1) and 25.16% (5995) of non-anchor genes (23,828) are expressed. We further classified non-anchor genes into basal genes (with RNAPII or H3K4me3 peaks) and other genes (without RNAPII or H3K4me3 interactions or peaks) as control. For genes with detectable expression, the expression level of anchor genes is significantly higher than that of non-anchor genes (*P* < 2.2e−16, Wilcoxon test, Fig. 3c). It was also observed that gene pairs looped together tend to be expressed in the high expression level simultaneously in our test tissue (Fig. 3d and Supplementary Fig. 7a–c). Visual inspection of ChIA-PET data on a genome browser revealed that multiple pairs of interacting genes are interconnected to form complex chromatin domains. Our observations indicated that up to 12,749 genes expressed in seedlings were involved in chromatin loops with multiple genes, whereas 1335 genes interacted with genes to form single loops. To further infer promoter proximal region-linked chromatin interactions, we constructed a genome-wide network using PPI and visualized the top 100 sub-networks using Cytoscape software (Fig. 3e). We found that genes with higher degree, which is positively correlated with higher peak intensity, tend to be highly expressed and more frequently connected to other high degree genes (Fig. 3f). An example showed a cluster of genes on chromosome 10, in which the genes with higher expression levels (*Zm00001d026678*, *Zm00001d026680*, *Zm00001d026681*, FPKM ranging from 26.449 to 53.253) tended to have higher interaction degrees (Fig. 3h, i), whereas the genes with lower expression level (*Zm00001d026685* and *Zm00001d026683* with FPKM 8.578 and 0.46) showed lower interaction degrees. Spatial proximity of promoter-proximal regions of active genes prompted us to examine whether genes associated with PPI are co-regulated. We calculated the Pearson correlation coefficient of expressions between looping gene pairs as well as genes belonging to the interaction sub-network (Fig. 3g). Our results revealed that genes appear to be co-expressed compared to randomly selected gene pairs with similar distance distribution and same H3K4me3 or RNAPII peaks (*P* < 2.2e−16, *T*-test). These results suggested that chromatin loops may play important roles in active gene transcriptional regulation.

### Chromatin loops link eQTLs to their associated genes

**Fig. 4:**
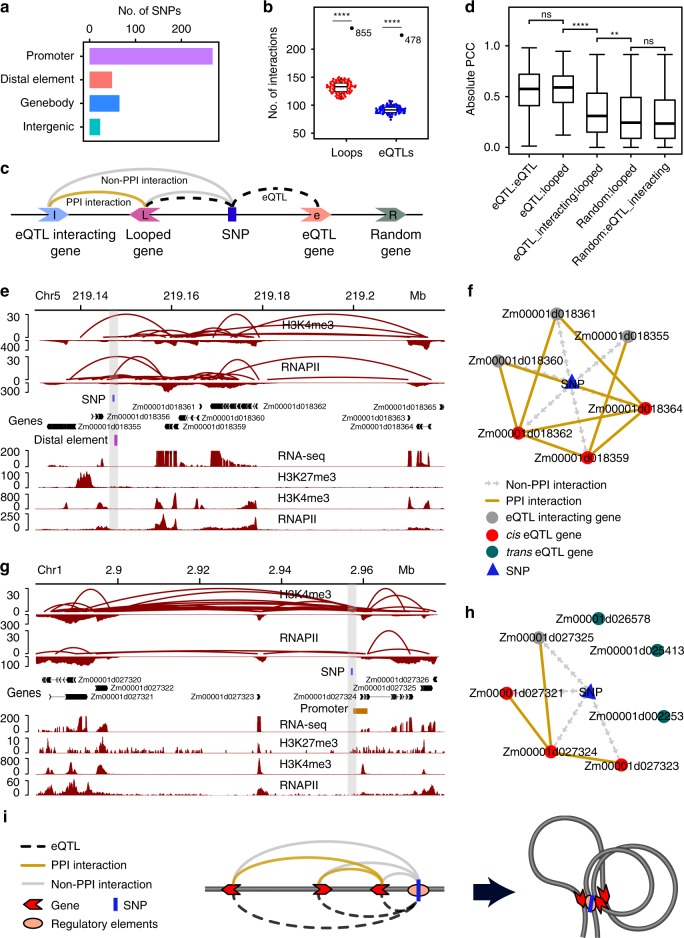
Regulation of eQTLs to their associated genes mediated by chromatin interactions. **a** Distribution of SNPs in promoter proximal regions, distal elements, gene bodies, and intergenic regions. **b** The numbers of overlapped interactions between ChIA-PET loops and eQTL SNP-eTrait pairs. The distance distribution of the simulated loops is similar to that from the ChIA-PET loops. ****P < 0.0001 from *T*-test. Values represent numbers of loops and eQTLs. **c** eQTL gene, genes that are associated with eQTL and not linked to eQTL by ChIA-PET loops; Looped gene, genes that are associated with eQTL and also linked to eQTL by ChIA-PET loops; QTL interacting gene, genes that are not associated with eQTL, but linked to an eQTL by ChIA-PET loops. Random gene, genes which are not associated with eQTL and not linked to eQTL by ChIA-PET loops. **d** The expression Pearson correlation coefficient of looped genes and random genes, eQTL interacting genes or eQTL genes. eQTL:eQTL represents the pairs of eQTL genes. eQTL:Loop represents the pairs of eQTL genes and Loop genes. eQTL_interacting:Loop represents the pairs of eQTL interacting genes and Loop genes. Random:Loop, the pairs of random genes and Loop genes. Random:eQTL_interacting, the pairs of random genes and eQTL interacting genes, random genes, the same distance distribution as the eQTL interacting genes and Loop genes. ****P < 0.0001, **P < 0.05, ns—*P* > 0.1 from Wilcoxon test. Boxplots show the median, and third and first quartiles. The whiskers are defined as 0.25–1.5 IQR/0.75 + 1.5 IQR, IQR is interquartile range. **e** A SNP in a distal element that affects the expression of multiple genes. **f** Interactions between genes potentially affected by distal elements SNP and other genes identified as expression QTLs in (**e**), PPI interaction means promoter proximal interaction. **g** A SNP in a promoter proximal region that affects the expression of multiple genes. **h** Interactions between genes potentially affected by a SNP in a promoter and other genes identified as expression QTLs in (**g**). **i** A proposed model based on chromatin interactions for eQTL regulation of gene expression. Source Data underlying (**b**, **c**) are provided in a Source Data file

In regard to long-range chromatin interactions, we examined the spatial relationship between eQTLs and genes associated with them. We collected 4691 eQTLs for 24,930 expression traits from a population of 105 intermated recombinant inbred lines derived from B73 and Mo17 (IBM population)^26^. The SNPs used for eQTL mapping in their study was called from RNA-Seq reads and only represents the transcribed regions of maize genome. We observed that 63.17% of these SNPs are distributed in gene promoter proximal regions, and the rest in gene bodies and distal elements. Non-distal elements intergenic sequences contain few SNPs associated with expression traits (Fig. 4a). Within a 4-kb window centered on top SNPs in the testable eQTLs, we detected 405 eQTLs that were involved in chromatin interactions with 475 genetically associated genes (eQTL–etrait gene pairs) (Fig. 4b). This number of eQTL–etrait gene pairs looped by chromatin interactions was significantly higher than the corresponding regions connected by the simulated ChIA-PET data with similar features, as a random control (Fig. 4b), indicating eQTL–etrait gene pairs tend to be spatially proximal to each other for potential regulation of gene expression. In addition, we identified 524 genes that looped with eQTL regions, but were not genetically associated with eQTL. These genes were referred to as eQTL interacting genes (spatial chromatin interaction with eQTL) (Fig. 4c). It was found that the expression of eQTL interacting genes and looped genes within *cis* eQTLs was strongly correlated to that of interacting sites, compared to that of random sites in the IBM population (*P* = 0.0019, Wilcoxon test; Fig. 4d). We thus speculated that eQTL interacting genes that interact with eQTL genetically associated genes may potentially be indirectly regulated by eQTLs. An example is an eQTL harboring a distal element interacting with 6 genes, including 3 eQTL associated genes, *Zm00001d018359*, *Zm00001d018362*, *Zm00001d018364* (Fig. 4e). In addition, 3 eQTL interacting genes (*Zm00001d018360*, *Zm00001d018361*, *Zm00001d018355*) are connected to eQTL associated genes via PPI interactions (Fig. 4e, f). Another example showed that, although an eQTL is associated with 3 *cis* eQTL genes, *Zm00001d027321*, *Zm00001d027323*, *Zm00001d027324*, and 3 *trans* eQTL genes, *Zm00001d026578*, *Zm00001d025413*, *Zm00001d002253*, interactions could be detected only between *cis* eQTL genes (Fig. 4g, h). In addition, another *cis* eQTL interacting gene *Zm00001d027325* was identified according to its PPI with *cis* eQTL genes. No *trans* eQTL associated genes were involved in chromatin interactions with the *cis* eQTL interacting genes observed, suggesting the presence of complex regulation between chromatin architecture, genetic variations, and gene expression. Based on these results, we propose a model, in which the chromatin interactions may provide cues to develop a topological framework between eQTLs and their associated genes (Fig. 4i), indicating a potential mechanism by which genetic variation impacts gene expression.

### Proximal–distal loops associated with phenotype variation

**Fig. 5:**
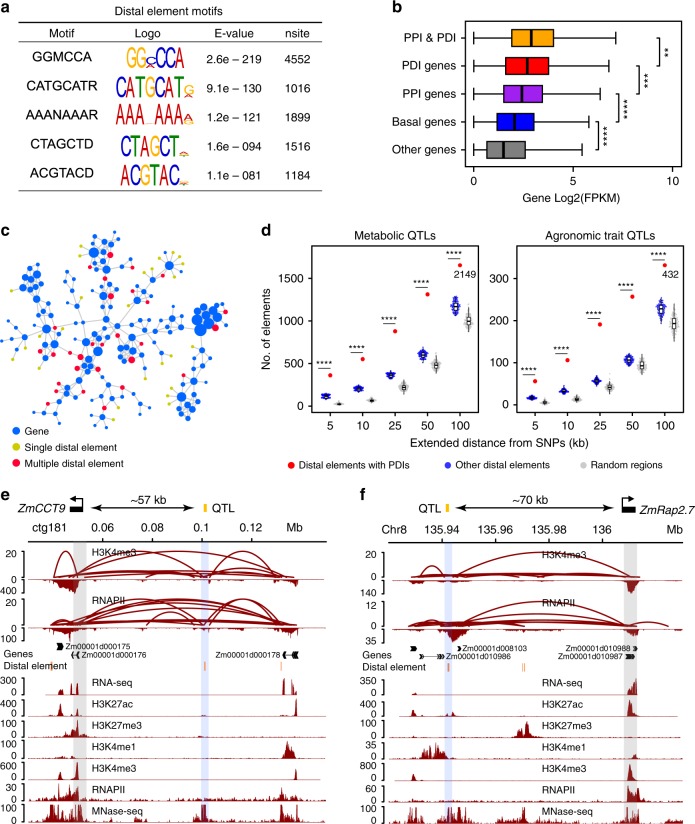
Chromatin loops connect QTLs to the genes causing phenotypic changes. **a** The five most common motifs from distal elements. **b** Expression analysis of genes (with FPKM > 1) involved in PDI and PPI (*n* = 4571), PDI genes (*n* = 621), PPI genes (*n* = 10,705), basal genes (*n* = 4959), and other genes (*n* = 877). Basal genes: genes containing H3K4me3 or RNAPII peaks but not involved in chromatin interaction; Other genes: genes neither marked with H3K4me3 or RNAPII peaks nor engaged in chromatin loops. ****P < 0.0001, ***P < 0.01, **P < 0.05 from Wilcoxon test. Boxplots show the median, and third and first quartiles. The whiskers are defined as 0.25–1.5 IQR/0.75 + 1.5 IQR, IQR is interquartile range. **c** Clusters of single distal elements and multiple distal elements (single distal elements represent those distal elements interacting with one gene, multiple distal elements represent those distal elements interacting with multiple genes). **d** Number of interaction distal elements detected at the indicated distance from single nucleotide polymorphisms (Other distal elements: distal elements without interactions. Random regions: random regions from genome). ****P < 0.0001 from *T*-test. Values represent numbers of distal elements and random regions. **e** Chromatin interactions and ChIP-Seq profiles at *ZmCCT9* (Zm00001d000176). **f** Chromatin interactions and ChIP-Seq profiles at *vgt1* (Zm00001d010987). Source Data underlying (**b**, **d**) are provided in a Source Data file

In maize, many QTLs in intergenic regions were identified to influence phenotypes by regulating the expression of target genes^16,18,20,22^. Since most distal elements are also intergenic, we tested whether long-range chromatin interactions may spatially link distal elements located in QTLs to target genes and thereby contribute to phenotypic variations. We found that distal elements were distributed mainly at both ends of a chromosome (Supplementary Fig. 8a), which are also enriched with active histone modifications, while DNA methylation is lower (Supplementary Fig. 8b). We identified certain conserved DNA motifs in distal elements, and that GGCCCA motif was the most common (Fig. 5a), in line with previous reports^4^. By overlapping chromatin interactions with distal elements and promoter proximal regions, we identified 9152 promoter-proximal and distal interactions (PDI) (Supplementary Data 5) involving 6293 distal elements (Supplementary Data 4). We found that genes involved in PDI were more abundantly expressed than genes involved only in PPI, followed by basal genes (Fig. 5b). Strikingly, 2567 distal elements interact with multiple genes (as multiple distal elements), while the other 3726 distal elements interact only with one gene (as single distal elements) (Fig. 5c). We speculate that the former might be involved in the regulation of multiple genes, while the latter may act as regulatory elements of single associated genes.

In order to examine the global nature of chromatin interactions in genetic regulation of complex traits, we selected the distal elements that are at least 1 bp overlapped to candidate region from GWAS studies by scanning up to a 200 kb window centered on the SNPs (Fig. 5d). We found that 2149 distal elements involving in chromatin interactions, comprising 1276 multi-distal elements and 873 single-distal elements, were overlapped with metabolic QTLs data^27^, indicating that such distal elements were potentially associated with metabolic traits. In addition, we identified that 290 multi-distal elements and 142 single-distal elements were overlapped with agronomic traits QTLs^28^, indicating that these distal elements were associated with agronomic traits through loop formation. Statistical analysis showed that the distal elements with interactions were significantly enriched at the QTL regions compared to the distal elements without interactions and random regions of the genome. For instance, a QTL regulating maize flowering time was mapped to a Harbinger-like transposable element 57 kb upstream of *ZmCCT9*, a CCT transcription factor. This Harbinger-like element repressed *ZmCCT9* in *cis* to promote flowering during long days, where the locus itself is diurnally regulated to suppress the florigen *ZCN8*, resulting in late flowering during long days^22^. We have now detected a pair of interaction anchors at the *ZmCCT9* promoter and at distal elements, respectively (Fig. 5e). Vegetative to generative transition 1 (*vgt1*) is another major QTL that controls flowering in maize^20,21^. A non-coding regulatory element *vgt1*, a repressor of flowering, was mapped 70 kb upstream of *ZmRap2*.*7*. We found that this noncoding regulatory element is 3 kb away from distal element interacting with *ZmRap2*.*7* frequently (Fig. 5f). These results indicate that distal elements potentially affect phenotypic variation by regulating the expression of the target genes through chromatin interactions. Considered together, these results may provide topological information regarding mechanisms underlying genetic regulation of complex traits.

## Discussion

In maize, coding regions only comprise a very small fraction of the whole genome, and the vast majority of the genome is non-coding regions. GWAS and QTL analysis have identified a lot of functional elements in maize, most of which are in the non-coding regions. So, it is important to study the regulatory elements in the non-coding regions and their target genes for better understanding of the biological mechanisms to the phenotypic traits.

In this study, we constructed the high-resolution three-dimensional structure of the maize genome with long-reads ChIA-PET method. We used formaldehyde to crosslink the tissues at first in order to fix the seedling quickly and followed by EGS fixation to further preserve protein-protein interactions that mainly mediate 3D genome organization. The method characterized the chromatin interactions between promoter proximal regions and distal regulatory elements, which provided potential functions for the regulatory elements through spatial connections. With the anchors in the length around 1 kb from ChIA-PET, our 3D genome map provides more detailed topological information compared to previous reports presenting high-order chromatin conformation using Hi-C data sets^15^.

In our study, we combined 3D genomics with histone modification, DNA methylation, gene expression, and chromatin accessibility. By quantifying histone modifications at transcription start sites, we observed a certain synergy between DNA methylation and histone modifications, where most genes marked with H3K27me3 are tissue-specific and the genes modified with H3K4me1 and H3K4me3 are mostly conserved. The data confirmed that epigenetic features associated with differentially expressed genes in different tissues.

Analysis of promoter proximal interactions revealed that majority of chromatin interactions occurred within chromosomes and indicated that each chromosome in the genome is an independent functional unit. Interacting genes can be identified via the chromatin interaction map and the genes interacting with higher numbers of other genes are also abundantly expressed. Furthermore, we found that the expression QTLs and eTraits are potential linked via chromatin interactions. Such analysis may explain spatially proximity between eQTL and eTrait and identify some potentially regulated genes, and provide a theoretical basis for gene expression regulation.

There are some limitations in eQTL and chromatin interactions analysis in this work. The first one is that the collected SNPs called from RNA-Seq reads, all of which are from transcribed regions. The paucity of SNPs in distal elements may be caused by the fact that relatively few of the distal elements are actually transcribed. Moreover, this analysis is limited by marker density and recombination frequencies in the population. Utilization of SNPs from whole genome resequencing would be more efficient to do such analysis in future.

The three-dimensional structure of a genome advances our understanding on the function of distal regulatory elements. Our approach revealed that thousands of genes interact with intergenic elements such as distal elements. Of note, some of these distal elements, like the distal elements which regulate *vgt1*, interact with only a single gene, while others, such as the distal elements which regulate *ZmCCT9*, interact with multiple genes. Notably, metabolic and agronomic QTLs are significantly enriched with distal elements, indicating that analyzing such elements may uncover the underlying functional sites. We also note that our approach is complementary to technologies such as Hi-C. However, our approach has a higher resolution for regulatory elements associated with chromatin interactions, and is easier for functional study for the protein used for chromatin immunoprecipitation. The approach and data presented in this study may provide a rich resource for maize functional genomic research, thus enhancing discourse on the genetic architecture associated with complex agronomic traits in crops.

## Methods

### Plant material and ChIA-PET libraries preparation

Seeds were germinated in the greenhouse at Huazhong Agricultural University, and seedlings were harvested 16 days after planting by cutting just above the soil line and preserving immediately in 1× PBS. Specimens were then fixed at room temperature with 1% (v/v) formaldehyde and vacuumed for 5 min. The vacuum was then turned off and released, and samples were further incubated for 25 min. Formaldehyde was quenched by adding glycine to a final concentration of 0.2 M. Subsequently, seedlings were rinsed thrice with deionized water, and incubated in 1.5 mM EGS for 45 min under vacuum. EGS fixative solution was prepared by dissolving 0.06 g EGS in 600 μL DMSO at 37 °C for 5 min, mixed with 89.4 mL 1× PBS, and incubated at 37 °C before use. Cross-linked samples were rinsed thrice with deionized water and immediately frozen in liquid nitrogen and stored at −80 °C. About 3–5 g of samples were ground in liquid nitrogen into fine powder and resuspended in 100 mL of EB1 buffer (0.4 M sucrose, 10 mM Tris–HCl, 5 mM b-mercaptoethanol and 1 mM PMSF). The mixture was filtered through Miracloth, and the filtrate was centrifuged at 1800×*g* for 10 min at 4 °C. The pellet was washed three times in 5 mL of EB2 buffer (0.25 M sucrose, 10 mM Tris–HCl, 5 mM b-mercaptoethanol, 10 mM MgCl_2_, 1% Triton X-100, and 1 mM PMSF) and centrifuged at 2000×*g* for 10 min at 4 °C. Next, the pellet was washed in 2 mL of EB3 buffer (1.7 M sucrose, 10 mM Tris–HCl, 5 mM b-mercaptoethanol, 2 mM MgCl_2_, 0.15% Triton X-100, and 1 mM PMSF) and centrifuged at 2000×*g* for 1 h at 4 °C. The final pellet was resuspended in 1 mL of NLB buffer (10 mM Tris–HCl, 20 mM EDTA, 400 mM NaCl, 1% Triton X-100, and 2 mM PMSF). The chromatin extract was fragmented into 1–3 kb by sonication using a Bioruptor (Diagenode) under HIGH intensity (30 cycles of 30 s ON and 50 s OFF). After centrifugation at 2000×*g* for 10 min at 4 °C, the supernatant was transferred to a new tube for chromatin immunoprecipitation (ChIP). To prepare antibody-loaded beads, 60–100 μL antibody (RNAPII, BioLegend cat. no. 920102 and H3K4me3, ABclonal cat. no. A2357) was mixed with 800 μL suspended protein G magnetic beads (~1:100 dilution) followed by incubation at 4 °C for 8 h with rotation. Then, mixed fragmented chromatin extract with antibody-loaded beads and incubated it at 4 °C for overnight with rotation. The supernatant was discarded and the beads were washed using 5 mL 0.1% SDS FA cell lysis buffer (0.05 M, HEPES-KOH, 0.15 M NaCl, 0.001 M EDTA, 1% Triton X-100, 0.1% sodium deoxycholate, and 0.1% SDS) for three times, then followed washing with 5 mL High salt ChIP buffer (0.05 M, HEPES-KOH, 0.35 M NaCl, 0.001 M EDTA, 1% Triton X-100, 0.1% sodium deoxycholate, and 0.1% SDS) twice; the beads were washed with 5 mL ChIP wash buffer (0.01 M Tris–HCl, 0.25 M LiCl, 0.001 M EDTA, 2.5% NP-40, and 0.5% sodium deoxycholate) once and finally followed washing with 5 mL 1× TE buffer (pH 8.0, Ambion, cat. no. AM9849) twice. ChIP DNA on beads was used for end-repair and A-tailing using T4 DNA polymerase (Promega, cat. no. M421F) and Klenow enzyme (NEB, cat. no. M0212L), the ChIP DNA ends were proximity-ligated by the single biotinylated bridge-linker, Forward strand: 5′-[5Phos]CGCGATATC/iBIOdT/TATCTGACT-3′, Reverse strand: 5′-[5Phos]GTCAGATAAGATATCGCGT-3′, with the 3′ nucleotide T over-hanging on both strands. Proximity ligation DNA was reverse cross-linked and fragmented before adding sequencing adaptors simultaneously by using Tn5 transposase (VAHTS; cat. no. TD501). DNA fragments contained the bridge-linker at ligation junctions were captured by Streptavidin beads, and used as templates for PCR amplification. These DNA products were then subjected to size-selection and paired-end sequencing (2 × 150 bp) using Illumina Hiseq X-Ten.

### Analysis of sequencing data

Several data sets used in this study are already published, including methylation^29^, and chromatin accessibility data^24^. RNA-Seq data from 16 days seedlings consistent with the ChIA-PET experiment. We removed the sequencing adapter and reads with low quality score from paired-end reads of ChIP sequencing, MNase sequencing, RNA sequencing, and bisulfite sequencing by Trimmomatic^30^. These clean reads were further aligned to the maize genome (B73 AGPv4) using BWA^31^, TopHat^32^, and BatMeth2^33^ with default parameters, respectively. Unmapped reads and non-uniquely mapped reads (mapping quality <30) were removed, while PCR duplicate reads were excluded by SAMtools^34^. To assess repeatability, we calculated coverage of the genome per 10 kb and normalized the coverage by reads per kilobase per million mapped reads (RPKM) using deepTools2^35^. Regarding profiles and heat maps surrounding transcription start sites, the coverage was generated with the genome binned at 50 bp. Based on the histone differences in the distribution of TSS regions of all genes on the maize genome, we divided them into 15 categories based on k-means cluster analysis. Read-enriched peaks were identified by MACS^36^. Among them, H3K4me3, H3K27ac (ABclonal, cat. no. A7253), and RNAPII peaks were identified with default parameters, whereas H3K27me3 (ABclonal, cat. no. A2363) and H3K4me1 (ABclonal, cat. no. A2355) were identified with parameters --broad -q 0.05. MNase sequencing peaks were identified with parameters --nomodel --shift -100 --extsize 200. Peaks were annotated using Homer^37^. RNA-Seq FPKM values were calculated with Cufflinks^38^. In order to calculate the methylation density for whole genome bisulfite DNA methylation, the total number of nucleotides C and T that overlapped with each genomic cytosine site across the whole genome was counted. The methylation level for each cytosine site was calculated by the sequencing depth of the site divided by the sum of unconverted and converted cytosines.

### Analysis of ChIA-PET data

ChIA-PET data were processed using modified ChIA-PET tools^39^, After filtering the linkers, the sequences were mapped to the maize genome (B73 AGPv4) using BWA. Unmapped reads, non-uniquely mapped reads (mapping quality <30) and PCR duplicates were removed. According to the genomic span between the two ends of a PET, each PET was categorized as a self-ligation PET, an inter-ligation PET or other PET. Self-ligation PETs were used to call binding peaks, whereas inter-ligation PETs were applied to call interactions. To obtain more robust chromatin interactions, detected interactions were included only if the paired-end tag count was ≥3 and at least one of the interacting anchors overlapped with one peak in corresponding ChIP sequencing data. Ultimately, 49,766 H3K4me3-mediated interactions were obtained, along with 25,002 interactions mediated by RNAPII. We transformed ChIA-PET unique mapping reads to contact frequency matrix and normalized the matrix based on distance among bins (resolutions are 1 Mb, 50 kb, 5 kb).

### Tissue-specific genes and housekeeping genes analysis

RNA-Seq data from 79 tissues^25^ were utilized to identify housekeeping genes and tissue-specific genes. We removed the sequencing adapter and reads with low quality score by Trimmomatic. The clean reads were aligned to the maize genome (B73 AGPv4) using TopHat with default parameters. Unmapped reads and non-uniquely mapped reads (mapping quality <30) were removed, while PCR duplicate reads were excluded by SAMtools. After merging data from the same tissue, RNA-Seq FPKM values of the 79 tissues were calculated with Cufflinks. In any of the 79 tissues, genes with FPKM values exceeding 1 were considered as expressed. The number of tissues a gene is expressed in was used to define expression breadth. For each gene, we calculated a coefficient of variation (CV = *S*/*X*_mean_, where *S* is the standard deviation, and *X*_mean_ indicates the mean expression of the gene across all tissues). All these genes were ranked based on the coefficient of variation. The top 500 genes are regarded as housekeeping genes, while the bottom 500 genes are treated as tissue-specific genes.

### Identification of promoter proximal regions and distal elements

Candidate promoter proximal regions were defined as segments 2 kb upstream and 1.5 kb downstream from the transcription start sites of genes. After subtracting lightly digested with MNase sequencing data, 43,744 peaks from heavily digested with MNase sequencing data these were considered candidate distal regulatory elements. Segments that overlapped with the gene body were excluded, along with those that overlapped with fragments within 2 kb of the gene, or with H3K4me3 peaks. Ultimately, we obtained 17,157 distal regulatory elements that overlapped 1,109,957 low and unmethylated DNA regions (LUMRs). LUMRs were defined as low methylated regions and unmethylated regions using BatMeth2^33^. The threshold for the low methylated regions was CG < 0.2, CHG < 0.1, and CHH < 0.1 with binning 200 bp. To identify motifs in candidate distal regulatory elements, their sequences were analyzed using default parameters via MEME^40^.

### Identification of PPI and PDI

Distal elements and promoter proximal regions were mapped to ChIA-PET interactions using BEDtools^41^. PPIs were defined as loops between 2 anchors sharing overlap with the promoter proximal regions of different genes, while PDIs denoted one anchor overlapped with a distal element, and the other overlapped with a promoter proximal region. We obtained 22,875 PPIs and 9152 PDIs in total.

Genes involved in PPIs may also form clusters. A global network was built to depict chromosome–chromosome relationships based on the 100 most representative clusters which contained PPIs with paired-end tag counts greater than 4. Networks were visualized using Cytoscape^42^ and rendered by a self-organized layout algorithm, the Spring-Electric Layout.

### Co-expression analysis

Using RNA-Seq data from 79 tissues, we calculated the Pearson correlation coefficients for expression of all gene pairs involved in PPIs. As a control, we randomly selected the same number and similar distance distribution of gene pairs with those involved in PPIs. We took the logarithm and divided the genomic distance regions of the PPI gene pairs into 20 fragments and calculated the number of gene pairs in each fragment. For each of the 20 slices, we randomly selected a gene in the PPI gene set and performed the following screening. If there was a gene (in the PPI gene) set in a specific distance range near the gene, we found a pair of random genes that met the distance requirements. Otherwise, we switched to another gene until we selected the gene pair of the specified number and distance. If the generated random gene pairs were the same from the PPI gene pairs, it would be removed. Meanwhile, we randomly selected the same number of intra-chromosome gene pairs. Both random procedures were repeated 1000 times.

### Reporting summary

Further information on research design is available in the Nature Research Reporting Summary linked to this article.

## Supplementary Information

Supplementary Information

Reporting Summary

Description of Additional Supplementary Files

Supplementary Data 1–5

Source data

## Data Availability

Data supporting the findings of this work are available within the paper and its Supplementary Information files. A reporting summary for this Article is available as a Supplementary Information file. The sequence data are available at NCBI BioProject under accession number PRJNA541043. The source data underlying Figs. 1d, e, 3b–d, f, g, 4b, c, 5b, d and Supplementary Figs. 2a–d, 8a and 8b are provided as a Source Data File. Details of chromatin loops are presented in Supplementary Data 1–5. All other data generated and analyzed during the current study are available from the corresponding author upon reasonable request.
