# Supplementary material for: Chromatin interaction maps reveal genetic regulation for quantitative traits in maize
Source: Nat Commun. 2019 Jun 14;10:2632. doi: 10.1038/s41467-019-10602-5 (PMC6572838; doi:10.1038/s41467-019-10602-5)
Supplement: Supplementary file 1 — Supplementary Information [file 41467_2019_10602_MOESM1_ESM.pdf]

Chromatin interaction maps reveal genetic regulation for quantitative traits in maize

Peng *et al.*

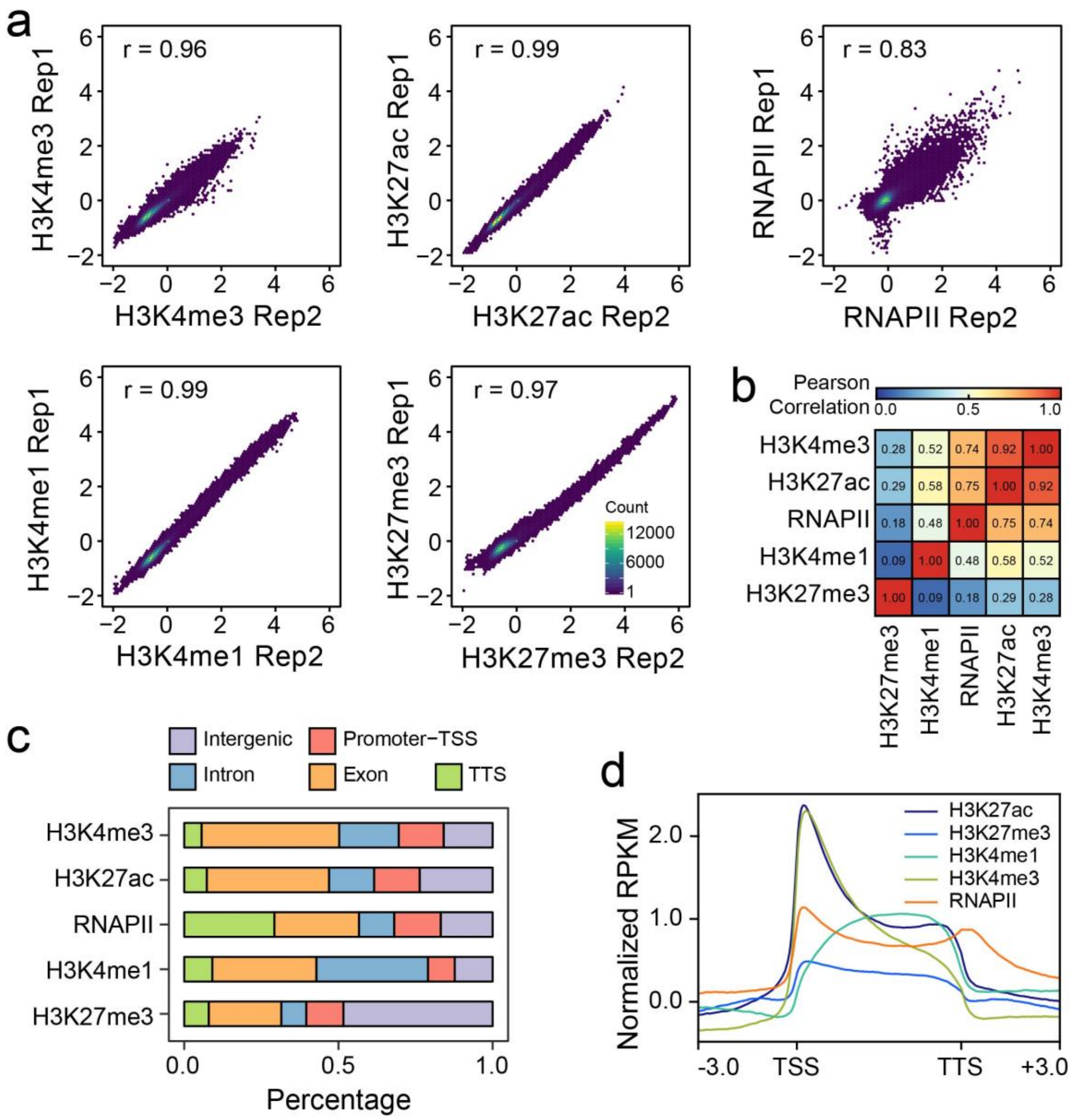

Supplementary Figure 1. Assessment of ChIP-Seq data reproducibility. a Scatter plots for replicates of sequence reads per 10 kb that are marked with indicated histone modifications and are occupied by RNAPII. Values are Pearson correlation coefficients (PCC). **b** Correlation between different histones. **c** Genomic distribution of histone modifications along gene model (TSS, transcription start sites; TTS, transcription termination sites). **d** RNAPII occupancy and histone modifications in genes (H3K4me1, H3K4me3, H3K27ac and H3K27me3; TTS for transcription termination sites).

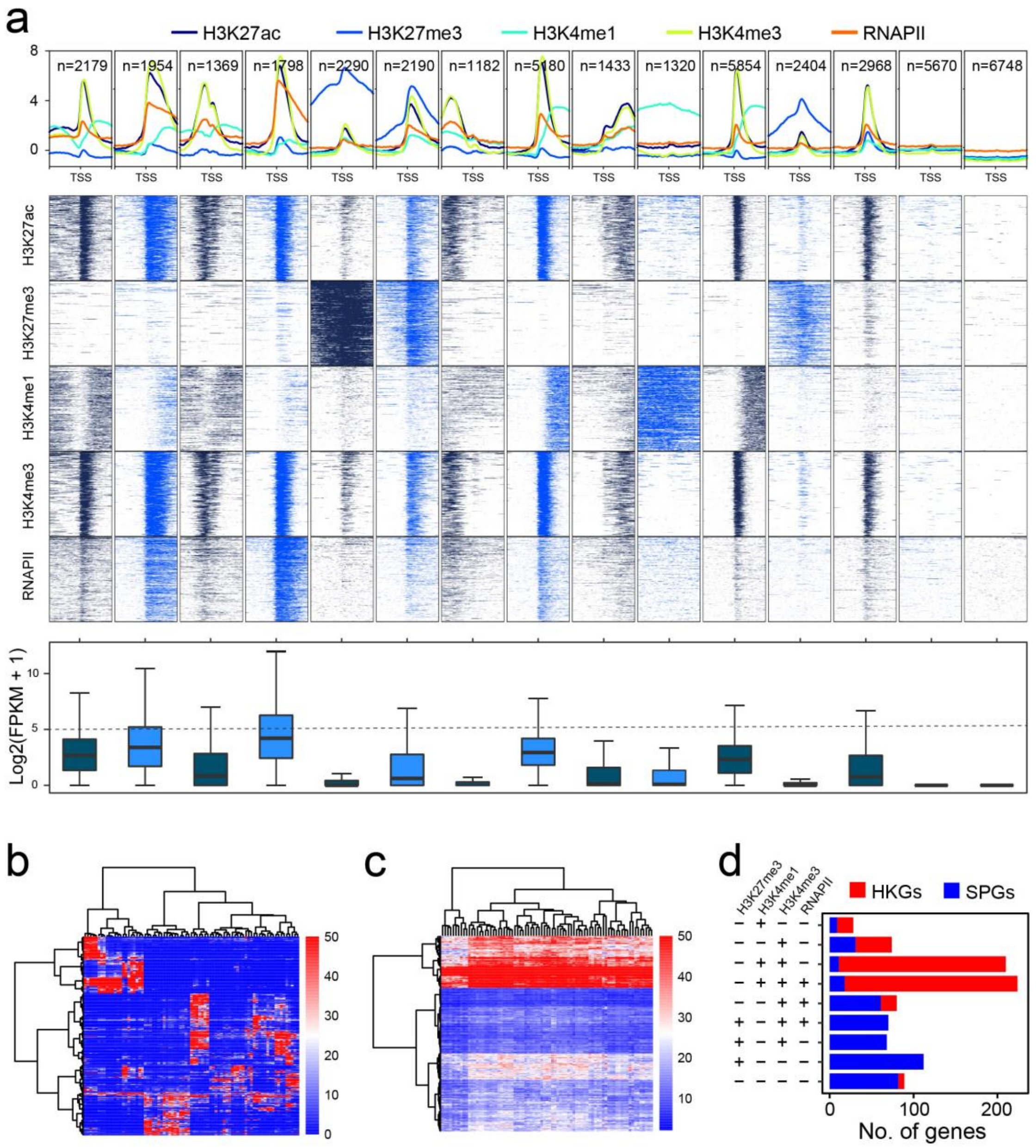

Supplementary Figure 2. Histone modification clusters associated with gene expression. a Heatmaps of different combinations of histone modifications and RNAPII occupancy at transcription start sites ± 3 kb. Boxplots represent expression levels of genes with similar combinations of epigenetic features. Boxplots show the median, and third and first quartiles. The whiskers are defined as 0.25 - 1.5 IQR / 0.75 + 1.5 IQR, IQR is Interquartile range. **b** Expression of tissue-specific genes in 79 tissues (each row represents a gene and each column represents one tissue). **c** Expression of housekeeping genes in 79 tissues (each row represents a gene and each column represents one tissue). **d** Distribution of tissue-specific gene expression and housekeeping gene expression in different combinations of histone modifications. Source data of Supplementary Figure 2a-d is provided as a Source Data file.

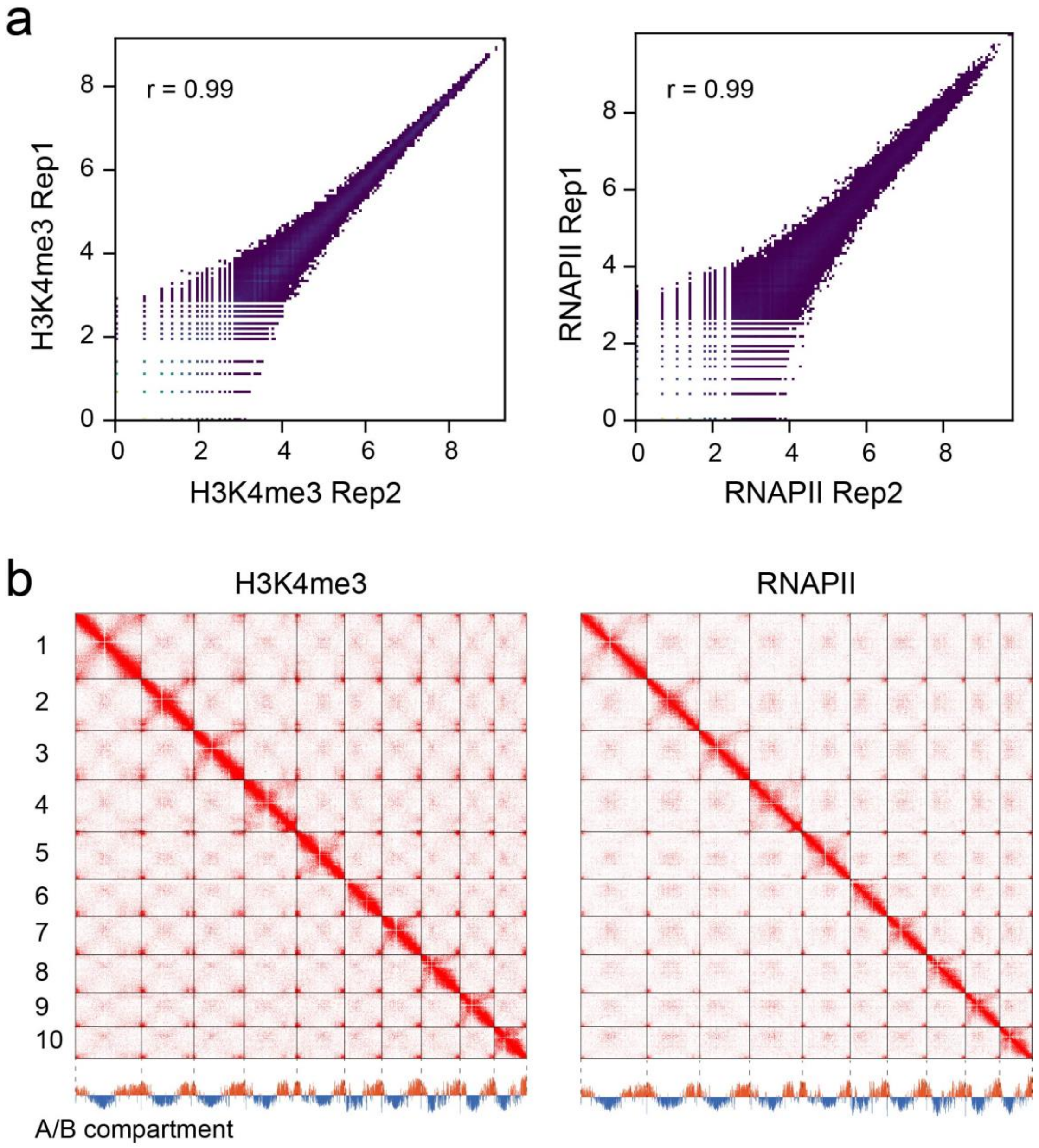

Supplementary Figure 3. ChIA-PET libraries reproducibility and heatmap of H3K4me3 and RNAPII occupancy in maize chromosomes. a Scatter plot between replicates of sequence reads per 10 kb that are marked with H3K4me3 and are occupied RNAPII. Values indicate Pearson's correlation coefficient (PCC). **b** Heat map of H3K4me3 and RNAPII occupancy in maize chromosomes.

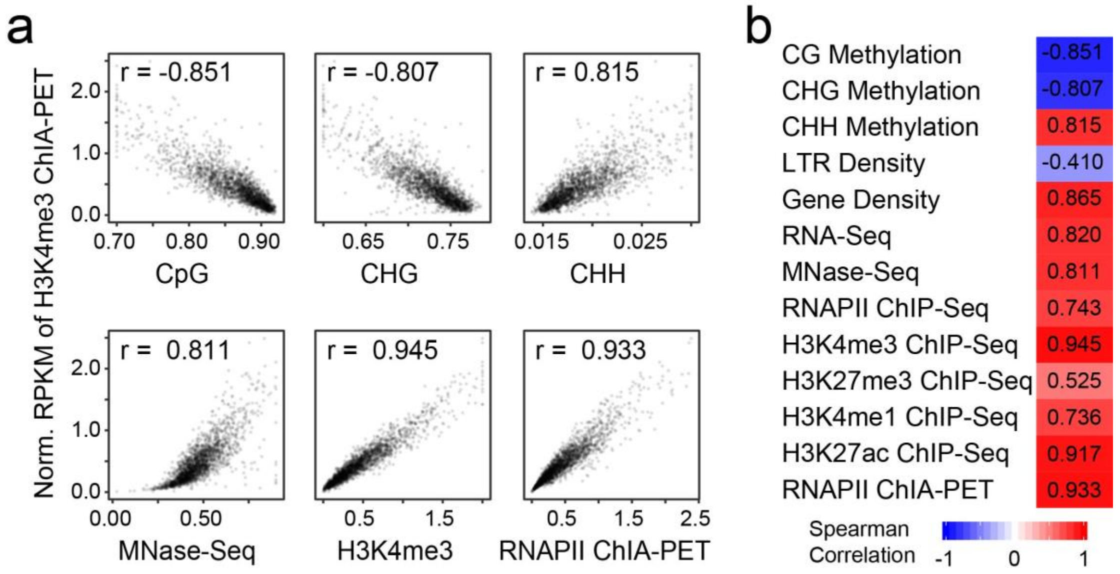

Supplementary Figure 4. Profiles of different epigenomic marks associated with ChIA-PET signals. Values are Spearman correlation coefficients. **a and b** Spearman correlation coefficients between the sequence reads per 1Mb from H3K4me3 ChIA-PET data and DNA methylation, RNAPII ChIA-PET data, and MNase regions.

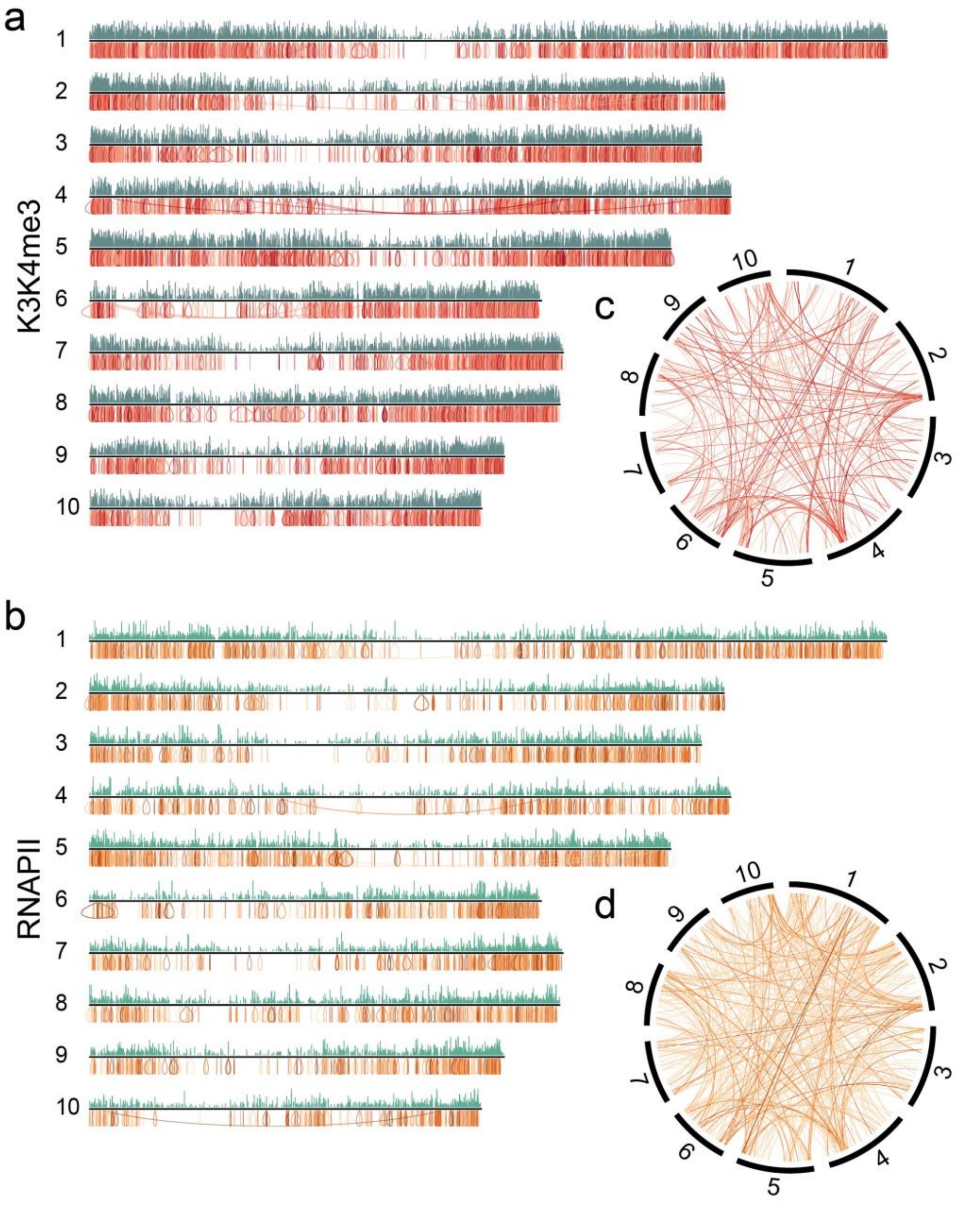

Supplementary Figure 5. Genome-wide view of ChIA-PET chromatin interactions. a and b Intrachromosomal loops (bottom) and peaks (top) inferred from H3K4me3 and RNAPII ChIA-PET library. Loop colors indicate intensity of contact frequency > 6, while peak height indicates intensity of RNAPII binding or H3K4me3 intensity. **c and d** Interchromosomal loop views of H3K4me3 and RNAPII ChIA-PET library. Loop colors indicate intensity of contact frequency > 6.

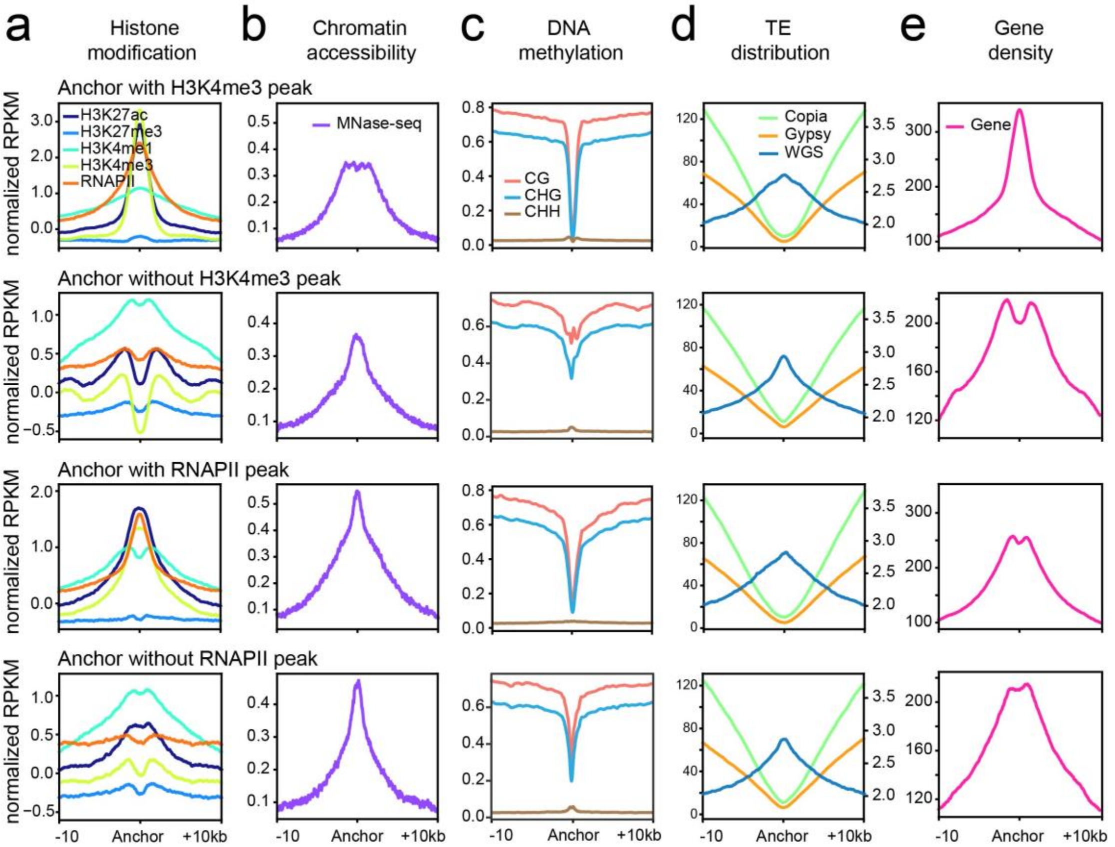

Supplementary Figure 6. Distributions of different epigenomic marks around loop anchors. From left to right, distribution of histone modification (**a**), chromatin accessibility (**b**), DNA methylation (**c**), TE content (**d**), gene density (**e**) on anchors with H3K4me3 peaks, and anchors without H3K4me3 peaks from H3K4me3 interactions, anchors with RNAPII peaks and anchors without RNAPII peaks from RNAPII interactions.

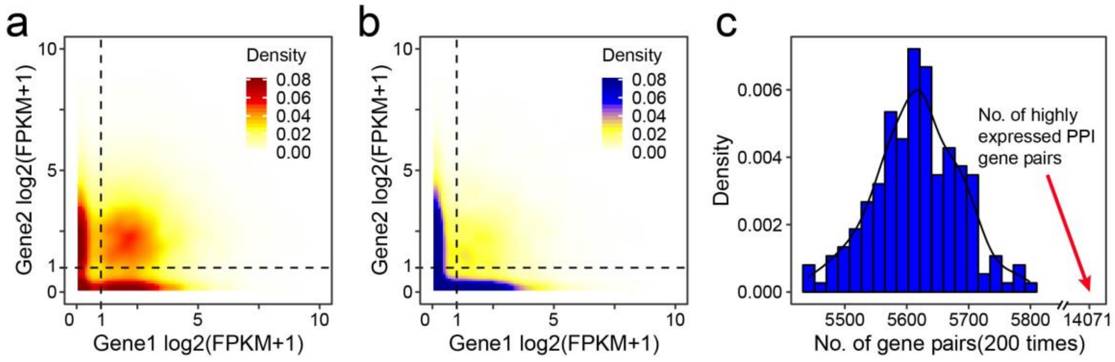

Supplementary Figure 7. PPI gene pairs are simultaneously highly expressed. Density plot of expressions from PPI gene pairs (**a**), density plot of expressions from one random simulation of gene pairs with similar distance distributions (**b**) and histogram of the numbers of gene pairs simultaneously highly expressed from 200 times of random simulation (**c**).

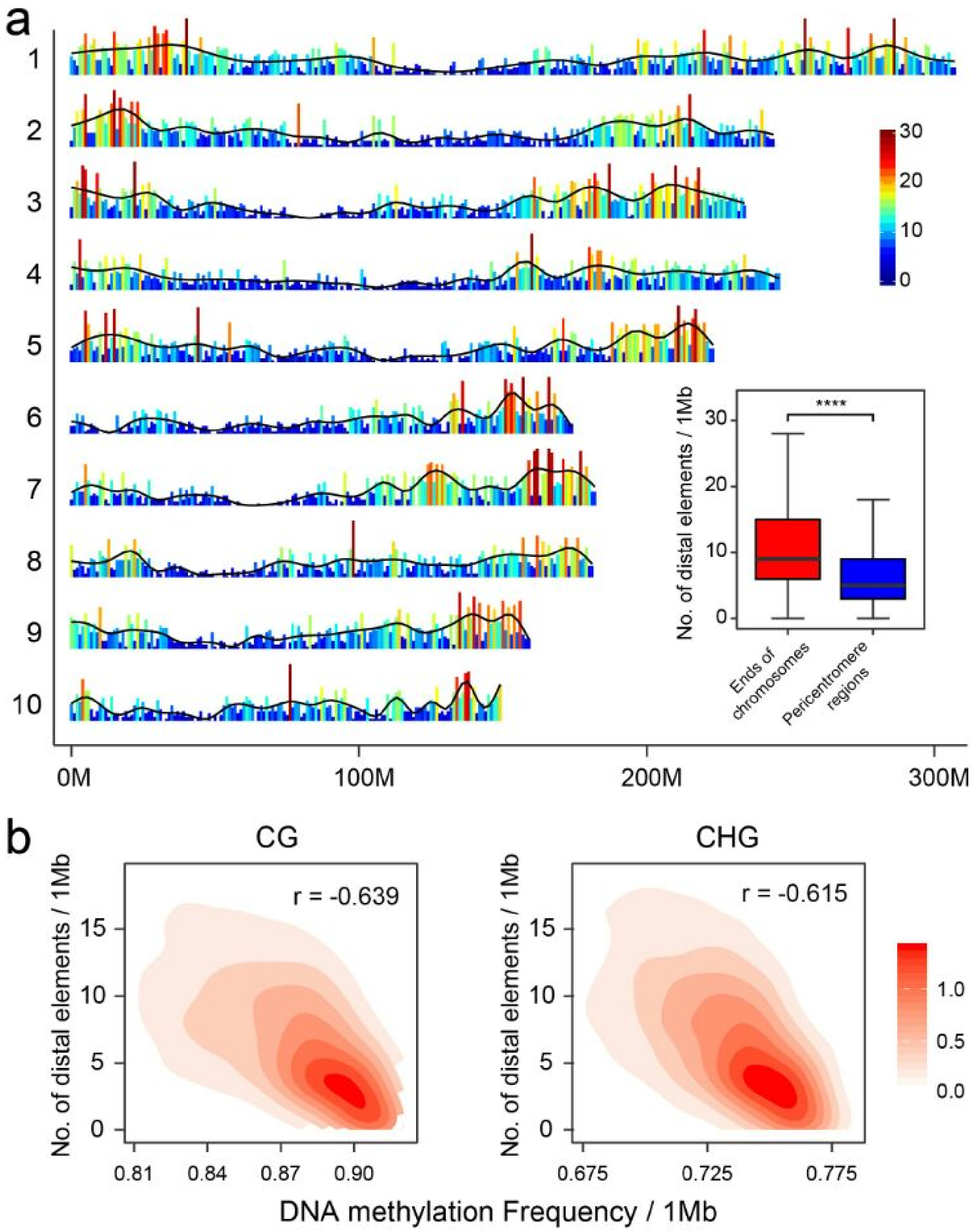

Supplementary Figure 8. The distribution of distal elements on chromosomes and association with DNA methylation. a Distribution of distal elements on each chromosome. The height of the bars represents the numbers of distal elements in the corresponding regions. Boxplots show the median, and third and first quartiles. The whiskers are defined as 0.25 - 1.5 IQR / 0.75 + 1.5 IQR, IQR is Interquartile range. **** for $P < 0.0001$ from Wilcoxon test. **b** Correlation

between distal elements and methylation at CG and CHG sites. Values are Pearson correlation coefficients. Source data of Supplementary Figure 8a and 8b is provided as a Source Data file.

Supplementary Table 1. Summary of ChIP-seq libraries*

Factor	Replicate	Total reads	Mapped reads	Uniquely Mapped Reads	Peak number	NSC	RSC
RNAPII	Rep1	40,974,962	36,379,544	25,469,227	12,947	1.18	0.85
RNAPII	Rep2	45,473,980	34,403,429	24,527,342	15,795	1.37	1.04
H3K4me1	Rep1	40,857,714	38,733,602	23,439,771	18,470	1.12	1.00
H3K4me1	Rep2	34,528,384	32,654,205	26,867,083	18,368	1.12	1.19
H3K4me3	Rep1	28,543,138	23,055,525	19,062,824	34,245	3.93	1.08
H3K4me3	Rep2	46,585,322	43,118,101	39,085,254	32,008	4.52	1.15
H3K27me3	Rep1	58,301,676	52,899,088	30,139,377	12,709	1.20	0.92
H3K27me3	Rep2	54,574,818	46,976,135	36,862,772	14,324	1.25	1.17
H3K27ac	Rep1	33,356,048	32,428,369	29,312,708	42,778	4.32	1.07
H3K27ac	Rep2	58,802,290	54,000,869	47,715,312	42,092	3.68	1.20

*Related to Figure 1.

Supplementary Table 2. Summary of ChIA-PET libraries*

Factor	Replicate	Total PETs	PETs with Linker	PETs with Linker ratio	Uniquely Mapped PETs	Self-ligation PETs	Self-ligation PETs ratio	Inter-ligation PETs	Inter-ligation PETs ratio	Clusters (PET≥3)	No. of intra-chromosome (PET≥3)
H3K4me3	Rep1	87,341,849	55,808,307	63.90%	19,914,795	5,883,339	29.54%	5,346,098	26.84%	25,574	24,996
H3K4me3	Rep2	108,183,640	72,873,824	67.36%	26,334,963	11,468,991	43.55%	5,636,999	21.41%	27,092	26,736
H3K4me3	Combined	195,525,489	128,682,131	65.81%	46,249,758	17,352,330	37.52%	10,983,097	23.75%	69,427	68,399
RNAPII	Rep1	87,742,460	54,305,537	61.89%	22,622,460	4,335,389	19.16%	1,854,687	8.20%	13,022	11,553
RNAPII	Rep2	82,952,645	51,906,831	62.57%	19,640,693	6,506,429	33.13%	3,650,770	18.59%	28,495	27,916
RNAPII	Combined	170,695,105	106,212,368	62.22%	42,263,153	10,841,818	25.65%	5,505,457	13.03%	52,043	49,895

*Related to Figure 2.
