# Supplementary material for: Chromatin interaction maps reveal genetic regulation for quantitative traits in maize
Source: Nat Commun. 2019 Jun 14;10:2632. doi: 10.1038/s41467-019-10602-5 (PMC6572838; doi:10.1038/s41467-019-10602-5)
Supplement: Supplementary file 2 — Reporting Summary [file 41467_2019_10602_MOESM2_ESM.pdf]

nature research

Corresponding author(s): Xingwang Li

Reporting Summary

Nature Research wishes to improve the reproducibility of the work that we publish. This form provides structure for consistency and transparency in reporting. For further information on Nature Research policies, see Authors & Referees and the Editorial Policy Checklist.

Statistical parameters

When statistical analyses are reported, confirm that the following items are present in the relevant location (e.g. figure legend, table legend, main text, or Methods section).

n/a	Confirmed	
☐	☒	The exact sample size (*n*) for each experimental group/condition, given as a discrete number and unit of measurement
☐	☒	An indication of whether measurements were taken from distinct samples or whether the same sample was measured repeatedly
☐	☒	The statistical test(s) used AND whether they are one- or two-sided<br>*Only common tests should be described solely by name; describe more complex techniques in the Methods section.*
☒	☐	A description of all covariates tested
☒	☐	A description of any assumptions or corrections, such as tests of normality and adjustment for multiple comparisons
☐	☒	A full description of the statistics including central tendency (e.g. means) or other basic estimates (e.g. regression coefficient) AND variation (e.g. standard deviation) or associated estimates of uncertainty (e.g. confidence intervals)
☐	☒	For null hypothesis testing, the test statistic (e.g. *F*, *t*, *r*) with confidence intervals, effect sizes, degrees of freedom and *P* value noted<br>*Give P values as exact values whenever suitable.*
☒	☐	For Bayesian analysis, information on the choice of priors and Markov chain Monte Carlo settings
☒	☐	For hierarchical and complex designs, identification of the appropriate level for tests and full reporting of outcomes
☐	☒	Estimates of effect sizes (e.g. Cohen's *d*, Pearson's *r*), indicating how they were calculated
☐	☒	Clearly defined error bars<br>*State explicitly what error bars represent (e.g. SD, SE, CI)*

Our web collection on statistics for biologists may be useful.

Software and code

Policy information about availability of computer code

Data collection	All sequencing libraries were prepared in house and raw reads were generated on Illumina high-throughtput sequencing paltform with manufacture's instruction. An expanded RNA-seq based B73 gene atlas (SRP010680) and WGBS data (SRR850328) and open chromatin data (SRP064243) are public data.
Data analysis	Software used include:Trimmomatic, BWA, Bowtie2, TopHat2, MACS2, deepTools2, BatMeth,as well as R version 3.4.3 (2017-11-30) and Python to run many of the mentioned programs. Detailed parameters of each of the programs are mentioned in relevant sections in Methods.

For manuscripts utilizing custom algorithms or software that are central to the research but not yet described in published literature, software must be made available to editors/reviewers upon request. We strongly encourage code deposition in a community repository (e.g. GitHub). See the Nature Research guidelines for submitting code & software for further information.

April 2018

Data

Policy information about availability of data

All manuscripts must include a data availability statement. This statement should provide the following information, where applicable:

- Accession codes, unique identifiers, or web links for publicly available datasets
- A list of figures that have associated raw data
- A description of any restrictions on data availability

All of raw data has been uploaded the sequencing data used in the manuscript to NCBI SRA with BioProject accession number PRJNA541043. (https://dataview.ncbi.nlm.nih.gov/object/PRJNA541043?reviewer=ub9hs4tqf3scoc9tjqeded49os).

Field-specific reporting

Please select the best fit for your research. If you are not sure, read the appropriate sections before making your selection.

☒ Life sciences ☐ Behavioural & social sciences ☐ Ecological, evolutionary & environmental sciences

For a reference copy of the document with all sections, see nature.com/authors/policies/ReportingSummary-flat.pdf

Life sciences study design

All studies must disclose on these points even when the disclosure is negative.

Sample size	No statistical methods were used to predetermine sample size.
Data exclusions	No exclusion of data was made.
Replication	All experimental data was reliably reproduced in multiple independent experiments as indicated in the figure legends.
Randomization	Randomization was not used.
Blinding	No blinding was used.

Reporting for specific materials, systems and methods

Materials & experimental systems

n/a	Involved in the study	
☒	☐	Unique biological materials
☐	☒	Antibodies
☒	☐	Eukaryotic cell lines
☒	☐	Palaeontology
☒	☐	Animals and other organisms
☒	☐	Human research participants

Methods

n/a	Involved in the study	
☐	☒	ChIP-seq
☒	☐	Flow cytometry
☒	☐	MRI-based neuroimaging

Antibodies

Antibodies used	Antibodies used for ChIP-seq: H3K4me1 polyclonal antibody (ABclonal, A2355), H3K4me3 polyclonal antibody (Millipore, 07-473; ABclonal, A2357), H3K27me3 polyclonal antibody (ABclonal, A2363), H3K27ac polyclonal antibody (ABclonal, A7253), and RNAPII monoclonal antibody (BioLegend, 920102)
Validation	The antibodies have been validated by the company using dot plot, western blot, immunofluorescence, ChIP-qPCR or ChIP-seq experiments. Please see information of antibodies below:<br>H3K4me1 antibody: https://www.abclonal.com.cn/catalog/A2355;<br>H3K4me3 antibody: http://www.merckmillipore.com/CN/zh/product/Anti-trimethyl-Histone-H3-Lys4-Antibody,MM_NF-07-473 or https://www.abclonal.com.cn/catalog/A2357;<br>H3K27me3 antibody: https://www.abclonal.com.cn/catalog/A2363;<br>H3K27ac antibody: https://www.abclonal.com.cn/catalog/A7253;<br>RNAPII antibody: https://www.biolegend.com/.

These commercial antibodies are well used and reported in many previous publications of our and other labs.

ChIP-seq

Data deposition

☒ Confirm that both raw and final processed data have been deposited in a public database such as GEO.

☒ Confirm that you have deposited or provided access to graph files (e.g. BED files) for the called peaks.

Data access links<br>*May remain private before publication.*	Data has been uploaded to NCBI SRA with BioProject accession number PRJNA541043. (https://dataview.ncbi.nlm.nih.gov/object/PRJNA541043?reviewer=ub9hs4tqf3scoc9tjqeded49os).
Files in database submission	18 data files including 10 epigenomic datasets and 4 ChIA-PET datasets (summarized in Extended Data Table S1 and Table S2) and ChIP-Seq INPUT data and 3 RNA-Seq data.<br>RNA-Seq_Rep1<br>RNA-Seq_Rep2<br>RNA-Seq_Rep3<br>Input_ChIP-Seq<br>RNAPII_ChIP-Seq_Rep1<br>H3K4me1_ChIP-Seq_Rep1<br>H3K4me3_ChIP-Seq_Rep1<br>H3K27me3_ChIP-Seq_Rep1<br>H3K27ac_ChIP-Seq_Rep1<br>RNAPII_ChIP-Seq_Rep2<br>H3K4me1_ChIP-Seq_Rep2<br>H3K4me3_ChIP-Seq_Rep2<br>H3K27me3_ChIP-Seq_Rep2<br>H3K27ac_ChIP-Seq_Rep2<br>RNAPII_ChIA-PET_Rep1<br>RNAPII_ChIA-PET_Rep2<br>H3K4me3_ChIA-PET_Rep1<br>H3K4me3_ChIA-PET_Rep2
Genome browser session<br>(e.g. UCSC)	Please use the following link: http://218.199.68.190:8008/basic/main/B73/ with username: "maize3d" and password: "maize_3d" to visualize peak files.

Methodology

Replicates	Two biological replicates for each histone mark (H3K4me3, H3K4me1,H3K27ac,H3K27me3),RNAPII in B73.
Sequencing depth	About 20 million pair-end (2x150bp) raw reads on average for each ChIP-seq experiment.
Antibodies	H3K4me1 polyclonal antibody (ABclonal, A2355), H3K4me3 polyclonal antibody (Millipore, 07-473; ABclonal, A2357), H3K27me3 polyclonal antibody (ABclonal, A2363), H3K27ac polyclonal antibody (ABclonal, A7253), and RNAPII monoclonal antibody (BioLegend, 920102)
Peak calling parameters	For narrow peak calling: macs2 callpeak function with "callpeak –t <input file> -c <control file> -f BAM –n <output peak file > -B -g 2.0e+9".For broad peak calling: broad-peak mode was used in MACS2 with FDR < 0.05, see Methods section in the manuscript for the details
Data quality	We used FRiP (fraction of reads in peaks), NSC (normalized strand coefficient) and RSC (relative strand correlation) to evaluate our data quality following Human ENCODE project guidelines. We also visualized peak signals on genome browser for each dataset. The number of narrow peak (FDR < 0.05, default parameter) is from 32,000 to 42,000; the number of broad peak (FDR < 0.05, default parameter) is from 12,000 to 18,000.
Software	MACS2, BWA, phantompeakqualtools, Bedtools, samtools, deepTools2 with details in Methods.
