# Supplementary material for: Chromatin interaction maps reveal genetic regulation for quantitative traits in maize
Source: Nat Commun. 2019 Jun 14;10:2632. doi: 10.1038/s41467-019-10602-5 (PMC6572838; doi:10.1038/s41467-019-10602-5)
Supplement: Supplementary file 3 — Description of Additional Supplementary Files [file 41467_2019_10602_MOESM3_ESM.docx]

**Description of Additional Supplementary Files**

File Name: Supplementary Data 1

Description: RNAPII mediated chromatin interaction clusters.

File Name: Supplementary Data 2

Description: Chromatin interaction clusters between H3K4me3-marked regions.

File Name: Supplementary Data 3

Description: Details of promoter proximal interactions (PPI).

File Name: Supplementary Data 4

Description: Candidate distal elements involved in promoter proximal-distal interactions.

File Name: Supplementary Data 5

Description: Details of promoter proximal-distal interactions (PDI).
